# Deep learning-based semantic segmentation for rice yield estimation by analyzing the dynamic change of panicle coverage

**DOI:** 10.3389/fpls.2025.1611653

**Published:** 2025-08-14

**Authors:** Hyeok-Jin Bak, Eun-Ji Kim, Ji-Hyeon Lee, Sungyul Chang, Dongwon Kwon, Woo-Jin Im, Woon-Ha Hwang, Jae-Ki Chang, Nam-Jin Chung, Wan-Gyu Sang

**Affiliations:** ^1^ National Institute of Crop and Food Science, Rural Development Administration, Wanju-gun, Republic of Korea; ^2^ National Institute of Horticultural and Herbal Science, Rural Development Administration, Muan-gun, Republic of Korea; ^3^ Department of Agronomy, Jeonbuk National University, Jeonju-si, Republic of Korea

**Keywords:** rice, phenotyping, deep learning, semantic segmentation, yield prediction, timeseries analysis, piecewise function

## Abstract

**Introduction:**

Rising global populations and climate change necessitate increased agricultural productivity. Most studies on rice panicle detection using imaging technologies rely on single-time-point analyses, failing to capture the dynamic changes in panicle coverage and their effects on yield. Therefore, this study presents a novel temporal framework for rice phenotyping and yield prediction by integrating high-resolution RGB imagery with deep learning-based semantic segmentation.

**Methods:**

High-resolution RGB images of rice canopies were acquired over two growing seasons. We evaluated five semantic segmentation models (DeepLabv3+, U-Net, PSPNet, FPN, LinkNet) to effectively delineate rice panicles. Time-series panicle coverage data, extracted from the segmented images, were fitted to a piecewise function to model their growth and decline dynamics. This process distilled key predictive parameters: *K* (maximum panicle coverage), *g* (growth rate), *d0* (time of maximum growth rate), a (decline rate), and *d1* (transition point). These parameters served as predictors in four machine learning regression models (PLSR, RFR, GBR, and XGBR) to estimate yield and its components.

**Results:**

In panicle segmentation, DeepLabv3+ and LinkNet achieved superior performance (mIoU > 0.81). Among the piecewise function parameters, K showed the strongest positive correlation with Yield and Grain Number (GN) (*r* = 0.87 and *r* = 0.85, respectively), while *d0* was strongly negatively correlated with the Filled Grain Ratio (FGR) (*r* = -0.71). For yield prediction, the RFR and XGBR models demonstrated the highest performance (R_2_= 0.89). SHAP analysis quantified the relative importance of each parameter for predicting yield components.

**Discussion:**

This framework proves to be a powerful tool for quantifying rice developmental dynamics and accurately predicting yield using readily available RGB imagery. It holds significant potential for advancing both precision agriculture and crop breeding efforts.

## 1 Introduction

Agricultural research now prioritizes improving sustainable productivity and efficiency to address the challenges posed by population growth and climate change. Data-driven agriculture supported by advanced technologies provides a novel means of reconciling productivity and environmental effects (Benti et al., 2024). Within data-driven agriculture, crop phenotyping via imaging techniques has received considerable interest. Phenotyping involves the detailed analysis of the morphological and physiological traits of crops, thereby informing variety selection, environmental adaptability assessment, and optimization of agricultural management (Jangra et al., 2021; Vishal et al., 2020).

Deep learning, particularly convolutional neural networks (CNNs), is fundamental to modern visual analytics. CNN, a type of deep learning model that uses convolutional kernels to extract features and classify images through multilayer neural networks (Alzubaidi et al., 2021), is highly effective at handling intricate visuals. CNNs are used for plant growth monitoring, pest detection, and yield prediction (Liu and Wang, 2021; Srivastava et al., 2022; Bak et al., 2024a, b). For major crops such as rice, quantitative analysis of the growth and yield components is essential for supporting food security and agricultural sustainability; deep learning provides an effective avenue to perform such evaluations (Kim et al., 2017).

Indeed, significant progress has been made in methods for detecting and quantifying rice panicles using these technologies. Foundational work has established high-quality public datasets for panicle segmentation (Wang et al., 2021), and object detection models, including advanced Vision Transformer-based architectures, have been widely applied for panicle counting (Wang et al., 2022; Wei et al., 2024; Lu et al., 2024). However, these counting-based methods face significant challenges as the canopy matures and panicles become occluded (Wang et al., 2022; Lu et al., 2024; Wei et al., 2024). An approach focusing on the total panicle area or coverage, rather than the count, may therefore offer a more robust signal. Yet, whether based on counting or area, these powerful methods predominantly rely on analysis at single or discrete time-points. While a few advanced studies have incorporated time-series analysis to track individual panicles (Zhao et al., 2017), a research gap persists in modeling the holistic dynamic change of the entire panicle canopy coverage with a continuous function.

To address this gap, this study develops an integrated framework. First, we leverage semantic segmentation to analyze panicle coverage, a technique well-suited for area-based analysis of complex crop structures (Lei et al., 2024; Madokoro et al., 2022; Abourabia et al., 2024). We evaluated established models such as DeepLabv3+ and U-Net to ensure precise pixel-level data extraction (Liu and Wang, 2021). Second, building on the principle of function-based time-series modeling successfully used in other crops (Stepanov et al., 2022; Guo et al., 2021), we apply a piecewise function to quantify the unique growth and decline dynamics of the panicle coverage. Finally, the parameters derived from this function are used as inputs for machine learning models to accurately estimate yield. This complete framework provides a novel method for leveraging canopy dynamics for data-driven rice breeding and management.

## 2 Materials and methods

### 2.1 Image acquisition

Rice canopy images for panicle detection were gathered at the fields of the National Institute of Crop Science (NICS) in Wanju-gun, Republic of Korea, during the 2022 and 2023 growing seasons. High-resolution RGB images were acquired using two imaging systems: a fixed-position PTZ (Pan–Tilt–Zoom) camera (Hanwha Vision XNP-8300RW, South Korea) and a handheld camera (Sony DSC-RX0-M2, Japan). The experimental site and image acquisition equipment are shown in **Supplementary Figure S1**.

The PTZ camera, mounted on a tower at a fixed height of 5 m, was used for time-series imaging. While the camera has pan-tilt-zoom capabilities, these were used only for initial framing of the plot; for all subsequent data acquisition, the camera remained in a fixed position to record nadir RGB images at 3840 × 2160 pixels, ensuring consistent imaging geometry. The PTZ camera, using the Wisenet WAVE (Hanwha Vision, South Korea) software, recorded images twice daily at 09:00 and 16:00. In contrast, the handheld camera captured images between 10:00 AM and 12:00 PM. This dataset, collected in 2022 and 2023, was used solely for training and validating the deep learning-based semantic segmentation models.

For the handheld camera, a specific visual alignment protocol was implemented to minimize human-induced variability. The camera was wirelessly tethered to a smartphone using the ‘Imaging Edge’ mobile application for real-time monitoring of the field of view. Four poles were used to clearly mark the corners of the 1 m² target quadrat within each plot. During image acquisition, the operator manually adjusted the camera’s position until the on-screen auxiliary gridlines visually aligned with the four corner poles of the quadrat. This procedure was repeated for every shot between 10:00 AM and 12:00 PM to ensure that the camera’s height, distance, and near-nadir viewing angle were kept as consistent as possible, resulting in a highly consistent pixel resolution of the target area across all images.

The entire dataset, collected from four rice cultivars (Nampyeong, Shindongjin, Dongjin-1, and Saeilmi), was used solely for training and validating the deep learning-based semantic segmentation models.

### 2.2 Image preprocessing

**Figure 1:**
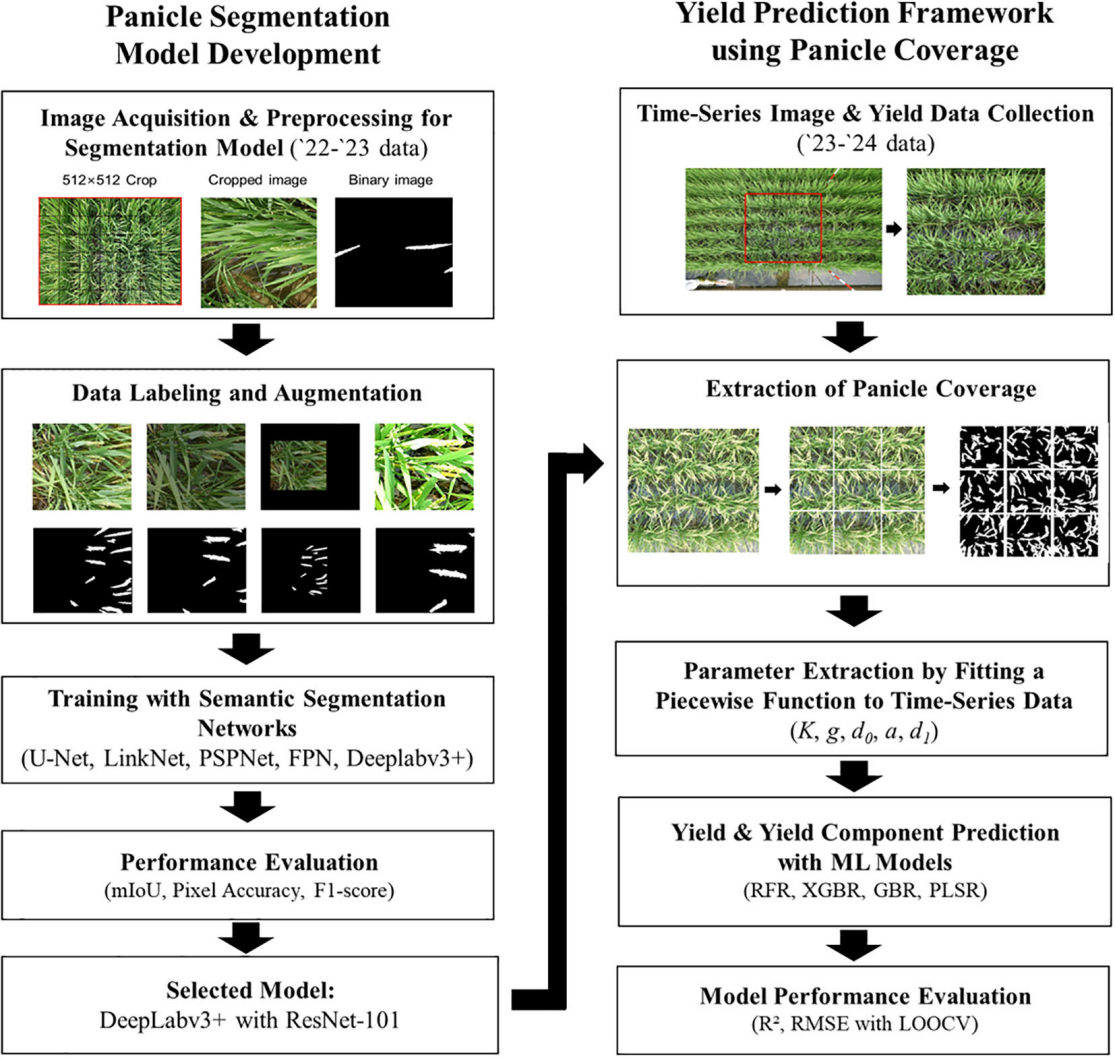
Panicle Segmentation Model Development and Yield Prediction Framework. Schematic overview of the entire research methodology. The left panel details the development pipeline for the deep learning-based panicle segmentation model, including image acquisition, data augmentation, training, and model selection using the ‘22-’23 dataset. The right panel illustrates the application of the selected model for time-series analysis and yield prediction using the ‘23-’24 dataset, covering panicle coverage extraction, parameter derivation, and machine learning-based prediction.

All acquired images underwent preprocessing for semantic segmentation training. OpenCV 4.9.0 (a Python-based image processing library) cropped the images to 512 × 512 pixels, improving computational efficiency and model performance. The rice panicle regions were then manually annotated as a single class using the LabelMe tool (Russell et al., 2008), after the heading stage. These annotations were converted into binary masks, in which 0 encoded background and 1 encoded panicles (**Figure 1**). Image augmentation techniques (Shorten and Khoshgoftaar, 2019), including resizing, Gaussian noise addition, and random brightness and contrast adjustments (**Figure 1**), were applied to improve model generalization and robustness. Specifically, the images were augmented with random brightness and contrast adjustments between 0.8 and 1.2, and were then upscaled (1.1–2.0) or downscaled (0.6–0.9) to vary size. This tripled the original dataset from 867 to 2,601 labeled images, producing greater diversity. This augmentation strategy simulated real-world variations in lighting, noise, and contrast, such as those caused by cloudy skies, shadows, and variable camera exposure, thereby enhancing model robustness at inference. Finally, the augmented dataset was divided into training, validation, and testing sets at a ratio of 7:2:1 to enable objective evaluation.

### 2.3 Deep learning architecture for rice panicle segmentation

This study evaluated the rice panicle segmentation performance with two backbone networks and five established semantic-segmentation architectures. The backbone networks, ResNet-50 and ResNet-101 (**Supplementary Table S1**), were selected for their capacity to extract hierarchical features and mitigate the vanishing gradient problem through residual learning. These pre-trained networks act as foundational feature extractors, providing rich, multi-scale representations essential for accurate pixel-level classification (He et al., 2016). The use of both ResNet-50 and ResNet-101 enabled comparison of the feature representation depth, with ResNet-101 potentially capturing finer details at the cost of increased computational resources. Five distinct semantic segmentation models were evaluated: DeepLabv3+ (Chen et al., 2018), U-Net (Ronneberger et al., 2015), PSPNet (Zhao et al., 2017), FPN (Lin et al., 2017), and LinkNet (Chaurasia and Culurciello, 2017) (**Supplementary Figure S2**). These models were selected to represent a range of architectural designs and feature-processing strategies commonly employed in pixel-level classification, particularly in agricultural image analysis. DeepLabv3+ was chosen for its capacity to capture long-range contextual information via atrous convolution and the Atrous Spatial Pyramid Pooling (ASPP) module. U-Net, with its encoder–decoder structure and skip connections, was included for its success in biomedical image segmentation and adaptability to diverse image analysis tasks. PSPNet, which utilizes a pyramid scene parsing network, was assessed to examine how global context affects segmentation accuracy. FPN was incorporated to evaluate the benefits of multi-scale feature representations for improved object delineation. Finally, LinkNet, known for its efficiency and real-time applicability, was used to explore the potential for computationally efficient segmentation. By systematically combining each of the five segmentation models with both ResNet-50 and ResNet-101, this study sought the optimal deep learning configuration for accurate and efficient rice panicle segmentation under field conditions.

Training parameters were tuned to standardize the input data and ensure stable learning. The image size was fixed at 512 × 512 pixels, the batch size was set to 8 to balance memory usage with optimization stability, and each model was trained for 200 epochs with a learning rate of 0.0001 to ensure gradual, stable improvement. Training was conducted on a system featuring an NVIDIA Quadro RTX 5000 GPU (16 GB), an Intel Xeon Gold 6226R CPU, and 256 GB of RAM. The operating system was Windows 11, with CUDA 12.5 for GPU acceleration, Python 3.9, and PyTorch 2.2.2 serving as the deep-learning framework (**Table 1**). Model validation and testing were conducted on the same system to ensure consistency.

**Table 1:** Training parameters, models, and hardware specifications used for the semantic segmentation tasks.

Category	Specification
Architectures and backbones	
Semantic segmentation models	DeepLabv3+, U-Net, FPN, LinkNet, PSPNet
Backbone networks used	ResNet-50, ResNet-101
Training hyperparameters	
Image input size	512 × 512 pixels
Batch size	8
Epochs	200
Optimizer	Adam
Learning rate	0.0001
System specifications	
CPU	Intel Xeon Gold 6226R
GPU	NVIDIA Quadro RTX 5000 (16 GB)
RAM	256 GB
Operating system	Windows 11
Framework and libraries	Python 3.9, PyTorch 2.2.2, CUDA 12.5

### 2.4 Evaluation of training accuracy

To evaluate the rice panicle detection performance, an independent evaluation dataset, separate from the training set, was used. Various metrics, including pixel accuracy, precision, recall, F1 score, and intersection over union (IoU), were used to assess the performance of the model comprehensively of the model comprehensively (Equations 1-5). Pixel accuracy denotes the proportion of correctly classified pixels among all pixels for evaluating the overall accuracy of the model. Precision is the proportion of actual panicle pixels among those predicted as panicles by the model, thus indicating the panicle prediction accuracy of the model. Recall is the proportion of actual panicle pixels correctly identified by the model; it is used to evaluate the panicle detection capability of the model. The F1 score, which is the harmonic mean of the precision and recall, combines both metrics. IoU is the ratio between the intersection area and the union area of the actual and predicted panicle regions, thus indicating the accurate segmentation ability of the model. These metrics are crucial to evaluating the rice panicle detection performance of the model from various aspects and determining its applicability to real-world environments. The equations for the metrics are as follows:


(1) $$\text{Pixel accuracy}=\frac{TP+TN}{TP+TN+FP+FN}$$

(2) $$\text{Precision}=\frac{TP}{TP+FP}$$

(3) $$\text{Recall}=\frac{TP}{TP+FN}$$

(4) $$\text{F}1\text{ score}=\frac{2}{\frac{1}{Recall}+\frac{1}{Precision}}$$

(5) $$\text{IoU}=\frac{TP}{TP+FP+FN}$$

where TP (true positive) denotes the number of pixels correctly classified as panicle, FP (false positive) denotes pixels incorrectly classified as panicle, FN (false negative) signifies panicle pixels incorrectly classified as background, and TN (true negative) represents background pixels correctly classified as background. These metrics offer a comprehensive evaluation of the overall segmentation performance of the models.

### 2.5 Experimental design for time-series panicle coverage analysis

To apply the trained segmentation models and build a yield prediction framework, separate time-series panicle coverage experiments were conducted in 2023 and 2024 at the National Institute of Crop Science (NICS), Republic of Korea, under both field and soil–bin conditions. Field transplantation occurred on June 7 and June 26, 2023, and on June 8, 2024. The transplantation on June 26, 2023, was designated late transplantation (LT). Soil bins (1 m × 1 m × 0.5 m) were placed outdoors, and transplantation occurred on June 9, 2023, and June 10, 2024. The primary cultivar was Nampyeong, with Dongjin-1, Shindongjin, and Saeilmi included in the 2024 experiments. Nitrogen was applied at three rates (0, 98.8, and 197.6 kg ha^-^¹), with treatments varying by year, environment (field or soil bin), and cultivar. Nitrogen was split into three doses following the Korean standard cultivation method in Korea: 50% as a basal dressing before transplantation, 20% as a tillering fertilizer 20 days after transplanting, and the final 30% as a panicle fertilizer at the panicle formation stage. Each experimental unit consisted of a 1 m² plot containing 28 hills. To analyze the relationship between the time-series panicle coverage and yield components, post-harvest measurements of the panicle number (PN), grain number (GN), number of grains per panicle (GNP), 1000-grain weight (TGW), and filled grain ratio (FGR) were conducted.

### 2.6 RGB image collection and preprocessing for yield component estimation

This section details the first step of our yield prediction framework: RGB image collection and preprocessing. The overall process, described in the following sections, involved (1) extracting panicle coverage from these images, (2) fitting the time-series data to a piecewise function to derive dynamic parameters (Section 2.7), and (3) using these parameters as inputs for machine learning models to predict final yield (Section 2.8). High-resolution RGB images were captured at intervals of 3–7 days throughout the growing season using a Sony DSC-RX0-M2 camera to document all key growth stages. For this study, which focuses on panicle coverage, the images taken from the heading onwards were used, as panicles are the primary subject of segmentation. The images were acquired between 10:00 AM and 12:00 PM, with the camera aimed at the center of the yield survey plot and leveled with the ground to minimize distortion. The captured RGB images were cropped to encompass a 1 m² area demarcated by the four corner poles within the plot, ensuring consistency in yield component measurements and reducing variability from inconsistent sampling. These cropped images were then resized to 1536 × 1536 pixels, nine times the model input size of 512 × 512 pixels, to ensure dataset uniformity (**Figure 1**). For the yield estimation study, an aggregate of 1,956 time-series images was acquired over multiple observation dates. These images were collected from 152 distinct plots (20 field plots in 2023, and 132 plots, including 80 field and 52 soil-bin plots in 2024). Panicle coverage was calculated by dividing each 1536 × 1536 image into nine 512 × 512 pixel tiles. The deep learning model then processed each sub-image to estimate the panicle coverage for that segment. The overall panicle coverage was determined by dividing the estimated panicle area by the total image area as (Equation 6).


(6) $$\text{PC}(\%)=\;\frac{\text{PA}}{\text{PA}+\text{BA}}$$

where PC denotes the panicle coverage (%), PA represents the panicle area of the image, and BA denotes the background area of the image. The estimated panicle coverage values from all nine segments were averaged to derive coverage per unit area (**Figure 1**). Representative resized images after the 2023 and 2024 treatments appear in **Supplementary Figure S3** to demonstrate the structure and quality of the dataset. Based on the performance evaluation presented in Section 3.2, the DeepLabv3+ model with a ResNet-101 backbone, which achieved the highest mIoU (0.82), was selected as the final model. This model was then used to segment all time-series images for the subsequent yield prediction analysis.

### 2.7 Fitting time-series data to a piecewise function

**Figure 2:**
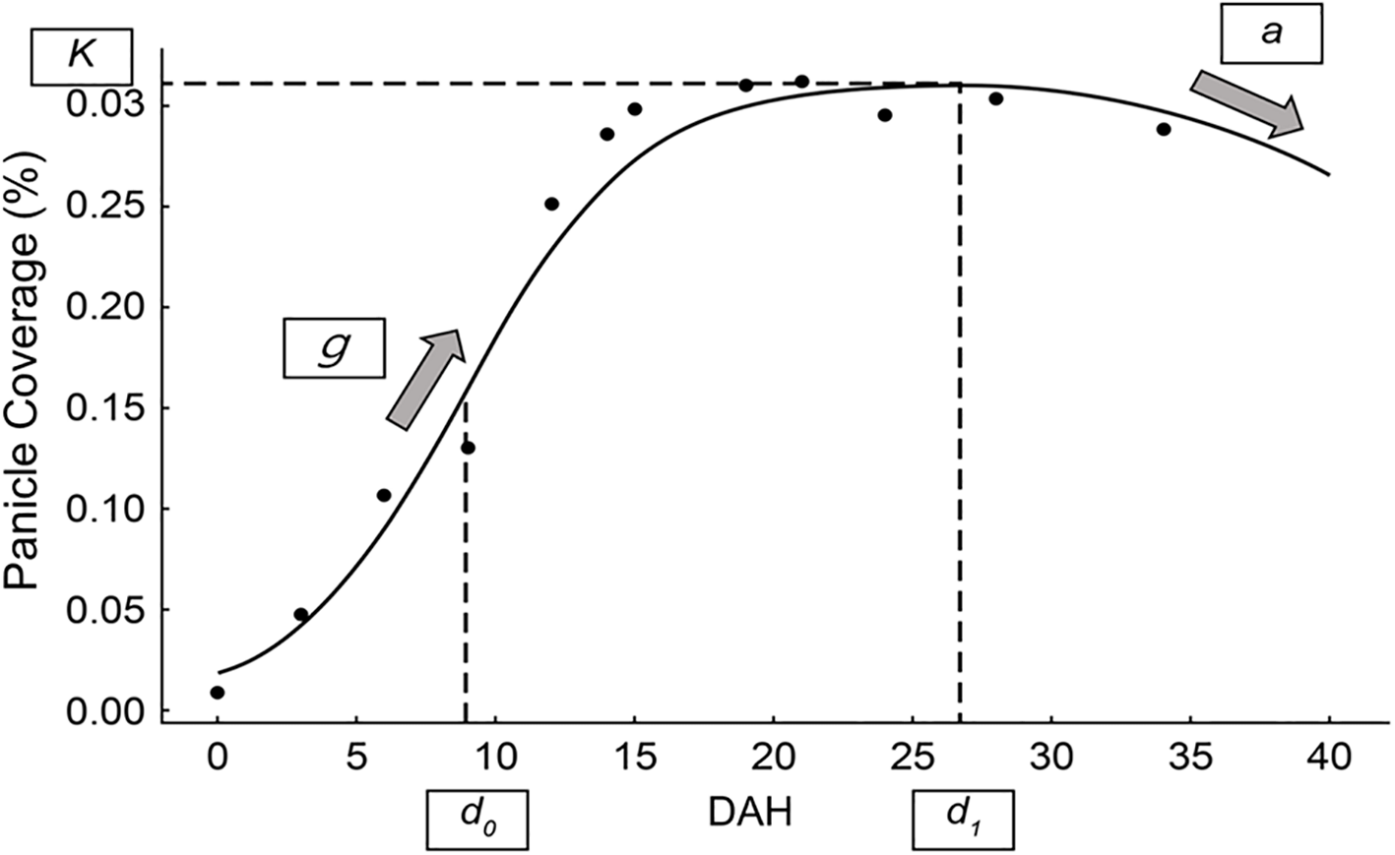
Piecewise function for modeling panicle coverage dynamics. A representative example of the piecewise function fit to time-series panicle coverage data. The figure illustrates the key parameters derived from the model: *K* (maximum panicle coverage), *g* (growth rate), *d_0_* (time of maximum growth rate), *a* (curvature of the decline phase), and *d_1_* (transition point between growth and decline phases).


The time-series panicle coverage data obtained from the segmented images were fit to a piecewise function (Equation 7) designed to model the growth and decline phases of the rice panicles, as shown in **Figure 2**. The function comprises a sigmoidal growth phase and a quadratic decline phase, seamlessly connected at the transition point. The function is expressed below.

(7) $$\text{f}(x)=\{\begin{array}{ll} \frac{\text{K}}{\left(1+{\text{e}}^{-\text{g}(\text{x}-{\text{d}}_{0})}\right)} & \;\text{if x}\leq {\text{d}}_{1}\\ \frac{\text{K}}{\left(1+{\text{e}}^{-\text{g}({\text{d}}_{1}-{\text{d}}_{0})}\right)}-\text{a}{\left(\text{x}-{\text{d}}_{1}\right)}^{2} & \;\text{if x}>{\text{d}}_{1} \end{array}\}$$

where *K* represents the maximum panicle coverage observed during the growth phase; *g* determines the growth rate, influencing the steepness of the sigmoidal curve. The parameter *d_0_* defines the point of maximum growth rate (inflection point), and *d_1_* marks the transition point between the growth and decline phases. Lastly, *a* controls the curvature of the quadratic decline, dictating how rapidly the panicle coverage decreases after *d_1_*. This piecewise function enables precise modeling of rice panicle dynamics, capturing the rapid increase during the heading stage and subsequent decline during senescence. The parameters derived from this model (*K, g, d_0_, d_1_*, and *a*) provide quantitative insights into panicle development under varying environmental and experimental conditions. These fitted parameters served to compare treatment effects on rice growth and yield. The parameters of this piecewise function for each experimental plot were determined by fitting the model to the time-series panicle coverage data using the non-linear least squares method. The optimization was performed using a scientific computing library (e.g., the curve_fit function from the SciPy library in Python). A key step in this process was providing robust initial guesses for the parameters to ensure stable convergence of the algorithm; these were estimated from the observed data trends for each plot. The goodness-of-fit for each resulting curve was then evaluated using the coefficient of determination (R²).

### 2.8 Machine learning model development for rice yield estimation

Machine learning models were developed for predicting the rice yield and its components using five parameters (*K*, *g*, *d_0_*, *a*, *d_1_*) extracted from a piecewise function fitted to the time-series panicle coverage data collected in 2023 and 2024. The models included partial least squares regression (PLSR), XGBoost regressor (XGBR), random forest regressor (RFR), and gradient boosting regressor (GBR).

The models were selected based on their documented performance in similar predictive modeling tasks in agricultural research. PLSR captures the linear relationship between the input and output variables, exhibiting stable predictive performance even under multicollinearity (Wold et al., 2001). RFR is an ensemble model that enhances the predictive performance by combining multiple decision trees (Breiman, 2001), whereas GBR and XGBR are boosting-based ensemble models that increase predictive accuracy by sequentially training weak learners (Friedman, 2001; Chen and Guestrin, 2016). The scikit-learn library serves primarily for model training and prediction.

Hyperparameter optimization was conducted for each model using GridSearchCV. Specifically, the PLSR model was set to n_components = 3, the RFR model with n_estimators = 100, and both the GBR and XGBR models with n_estimators = 100 and learning_rate = 0.05. The model performance was assessed using leave-one-out cross-validation (LOOCV). The root mean squared error (RMSE) and coefficient of determination (R²) served as evaluation as evaluation metrics (Equations 8, 9). Tree SHapley Additive exPlanations (Tree SHAP) analysis was performed to clarify the RFR and boosting-based models (XGBR, GBR). The RMSE equals the square root of the mean squared difference between the predicted and actual values, calculated as


(8) $$RMSE=\sqrt{\frac{1}{2}\sum_{i=1}^{n}{\left({\hat{y}}_{i}-y_{i}\right)}^{2},}$$

where $y_{i}$ denotes the actual values, ${\hat{y}}_{i}$ denotes the predicted values, and n is the number of data points. The coefficient of determination (R²) is the proportion of the variance in the actual values that is predictable from the predicted values, calculated as


(9) $${\text{R}}^{2}=1-\;\frac{\sum_{i=1}^{n}{\left(y_{i}-\hat{y}\right)}^{2}}{\sum_{i=1}^{n}{\left(y_{i}-\bar{y}\right)}^{2}}$$

where $\bar{y}$ represents the mean of the actual values.

### 2.9 Statistical analysis

To assess the effects of the experimental factors on the parameters derived from the piecewise function (*K*, *g*, *d_0_*, *a*, and *d_1_*), an analysis of variance (ANOVA) was performed using the statsmodels library in Python. Due to differences in the experimental design between the two years, the data for each year were analyzed separately. For the 2023 data, a one-way ANOVA was used to test the effect of the different treatment levels, which included nitrogen rates and transplantation dates. For the 2024 data, a three-way ANOVA was conducted to test the main effects of nitrogen, cultivar, and location, as well as their two-way interaction effects. All effects were considered statistically significant at *p* < 0.05.

### 2.10 Overall research framework

The comprehensive methodology, visually summarized in **Figure 1**, is structured into two main components. The left panel details the development and validation of the deep learning model for panicle segmentation, while the right panel illustrates how this trained model is subsequently applied within a time-series analysis pipeline to extract dynamic growth parameters and, ultimately, to predict rice yield and its components using machine learning regression.

## 3 Results

### 3.1 Model training and validation

The training and validation performance of five semantic segmentation models—DeepLabv3+, PSPNet, U-Net, FPN, and LinkNet—was evaluated using two different backbone architectures, ResNet-50 and ResNet-101. Both training loss and validation accuracy were monitored over 200 epochs to analyze the convergence trends (**Supplementary Figure S4**).

Across all models and backbones, the training loss consistently decreased, whereas the validation accuracy increased and subsequently stabilized as epochs increased. Models using ResNet-101 generally exhibited a slightly higher validation accuracy than ResNet-50, reflecting the enhanced feature extraction capabilities of the deeper backbone. Although the convergence patterns varied across the models, all achieved stable, low validation loss by the end of training. U-Net demonstrated the fastest initial stabilization, whereas other models, such as PSPNet, required more epochs to achieve similar final loss values.

This analysis confirms that all five segmentation models with both ResNet-50 and ResNet-101 backbones successfully converged, exhibiting decreasing training loss and stable validation accuracy. These results emphasize the effectiveness of these architectures in segmenting rice panicle images, with variations in convergence speed and final accuracy depending on the specific model and backbone combination.

### 3.2 Model performance comparison

To evaluate the performance of the models trained with two backbones (ResNet-50 and ResNet-101) and five semantic segmentation models (U-Net, FPN, LinkNet, PSPNet, and DeepLabv3+), the performance metrics were calculated using a test image set (**Table 2**). These metrics included the pixel accuracy, precision, recall, F1-score, and mean IoU (mIoU), offering a comprehensive overview of the segmentation quality and performance of each model.

**Table 2:** Performance comparison of semantic segmentation models.

Model	Backbone	Pixel accuracy	Precision	Recall	F1-score	mIOU
U-Net	ResNet-50	0.98	0.85	0.86	0.85	0.77
FPN		0.98	0.85	0.84	0.84	0.76
LinkNet		0.98	0.85	0.89	0.87	0.81
PSPNet		0.97	0.73	0.78	0.76	0.61
DeepLabv3+		0.99	0.87	0.88	0.88	0.81
U-Net	ResNet-101	0.98	0.88	0.84	0.86	0.78
FPN		0.98	0.87	0.86	0.85	0.78
LinkNet		0.99	0.86	0.90	0.88	0.81
PSPNet		0.97	0.72	0.77	0.73	0.59
DeepLabv3+		0.99	0.86	0.90	0.88	0.82

The table presents key performance metrics, including pixel accuracy, precision, recall, F1-score, and mean Intersection over Union (mIoU), for the five evaluated architectures using both ResNet-50 and ResNet-101 backbones.

Among the models with the ResNet-50 backbone, DeepLabv3+ attained the highest mIoU (0.81), F1-score (0.88), and pixel accuracy (0.99). LinkNet also scored well with an mIoU of 0.81 and an F1-score of 0.87. PSPNet had the lowest mIoU (0.61) among the models employing ResNet-50. Among the models with the ResNet-101 backbone, DeepLabv3+ again recorded the highest mIoU (0.82) and an F1-score of 0.88. LinkNet ranked second with an mIoU of 0.81 and an F1-score of 0.88. Across all models, the use of ResNet-101 generally resulted in a modest improvement in the recall and mIoU over that of ResNet-50, although the magnitude of this improvement varied.

A visual inspection of the segmentation results (**Supplementary Figure S5**) showed that despite these differences in the numerical metrics, the qualitative performance of panicle detection was generally high across all models. All models segmented the panicle regions, with DeepLabv3+ and LinkNet showing slightly better quantitative results, particularly in terms of the mIoU.

These findings indicate that although the numerical performance metrics highlight subtle differences between the models, practical model selection may depend on the computational efficiency, task-specific requirements, or hardware constraints rather than significant differences in the segmentation capability. DeepLabv3+ and LinkNet emerge as strong candidates due to their consistently high performance across both backbones. Future research should prioritize evaluating the robustness of these models across diverse datasets and environmental conditions to optimize their application to real-world tasks.

### 3.3 Analysis of parameter values by treatment

**Table 3** lists the fitted parameters (K, g, d_0_, a, d_1_) for various treatment conditions in 2023 and 2024. Through analysis of variance (ANOVA), we confirmed that factors such as nitrogen level, transplantation date, and rice variety had a statistically significant effect on the dynamics of panicle development, as quantified by these parameters (**Supplementary Table S2**).

**Table 3:** Fitted parameters of the piecewise function for rice panicle coverage under different treatment conditions in 2023 and 2024.

Year	Treatment	*K*	*g*	*d_0_*	*a*	*d_1_*	R^2^
2023	23-F-0N	0.2512	0.2738	9.9412	0.0003	28.0717	0.9858
	23-F-0N-LT	0.2081	0.2889	8.3847	0.0002	26.11	0.9825
	23-F-9N	0.306	0.2681	8.6695	0.0003	27.0125	0.9876
	23-F-9N-LT	0.3078	0.3712	7.7353	0.0001	25.7043	0.9958
	23-S-0N	0.3272	0.2658	9.5009	0.0003	28.9899	0.9926
	23-S-18N	0.3505	0.2649	8.2946	0.0003	27.4182	0.9932
	23-S-9N	0.3455	0.2781	8.3295	0.0003	27.0919	0.9936
2024	24-F-0N-DJ	0.2067	0.3683	8.5425	0.0002	26.3238	0.9932
	24-F-0N-NP	0.222	0.3098	10.311	0.0001	29.3876	0.9967
	24-F-0N-SD	0.1574	0.2746	11.0649	0	31.9771	0.9918
	24-F-0N-SI	0.211	0.2103	13.0975	0	34.027	0.9918
	24-F-9N-DJ	0.25	0.4606	4.9593	0.0002	17.093	0.9672
	24-F-9N-NP	0.2663	0.3667	7.5438	0.0002	23.7445	0.986
	24-F-9N-SD	0.2102	0.3355	7.8603	0.0001	25.8459	0.9902
	24-F-9N-SI	0.2782	0.2695	8.9365	0.0002	28.3735	0.9905
	24-S-0N	0.193	0.198	17.6353	0.0002	40.1652	0.9932
	24-S-9N	0.2293	0.2053	14.5728	0.0002	37.9843	0.9908
	24-S-18N	0.2171	0.206	16.2943	0.0003	38.9029	0.9923
ANOVA	Nitrogen	<0.001	<0.001	<0.001	5.10E-02	<0.001	
	Cultivar	8.66E-03	<0.001	<0.001	<0.001	<0.001	
	Transplanting	<0.001	<0.001	<0.001	8.33E-02	<0.001	

The parameters *K* (maximum panicle coverage), *g* (growth rate), *d_0_* (inflection point/time of maximum growth rate), *a* (curvature of the decline phase), and *d_1_* (transition point between growth and decline phases) were estimated for each treatment. R² values indicate the goodness-of-fit of the piecewise function. Treatment codes: F, Field; S, Soil–bin; 0N, 0 kg ha^-^¹ nitrogen; 9N, 98.8 kg ha^-^¹ nitrogen; 18N, 197.6 kg ha^-^¹ nitrogen; LT, Late transplantation; DJ, Dongjin-1; NP, Nampyeong; SD, Shindongjin; SI, Saeilmi. The number before the hyphen represents the year of the experiment. The ANOVA results at the bottom of the table show the significance levels (*p*-values) for the effects of nitrogen, cultivar, and transplanting factors on each parameter.

Across both years, *K* (maximum panicle coverage) generally increased with higher nitrogen levels, affirming the critical role of nitrogen. For instance, in the 2023 field experiment, *K* increased from 0.2512 under no nitrogen (23-F-0N) to 0.3060 with nitrogen addition (23-F-9N). The parameter *g* (growth rate) also tended to rise with nitrogen application in most cases, such as in the 2024 Nampyeong variety field experiment, where it rose from 0.3098 (0N) to 0.3667 (9N), indicating accelerated growth.

The timing of key developmental stages also shifted. The time of maximum growth rate (*d_0_*) advanced under higher nitrogen levels, shifting from 9.94 days (23-F-0N) to 8.67 days (23-F-9N) in the 2023 field data. Similarly, the start of the decline phase (*d_1_*) was advanced in parallel, occurring at 27.01 days compared to 28.07 days. The curvature of the post-peak decline (*a*) showed varied responses, though it marginally increased with higher nitrogen in some treatments.

The piecewise function demonstrated reliable performance in capturing panicle dynamics across diverse conditions, with the goodness-of-fit R² values remaining high, nearly all above 0.98.

### 3.4 Effects of nitrogen, transplantation date, and crop variety on panicle coverage dynamics

**Figure 3:**
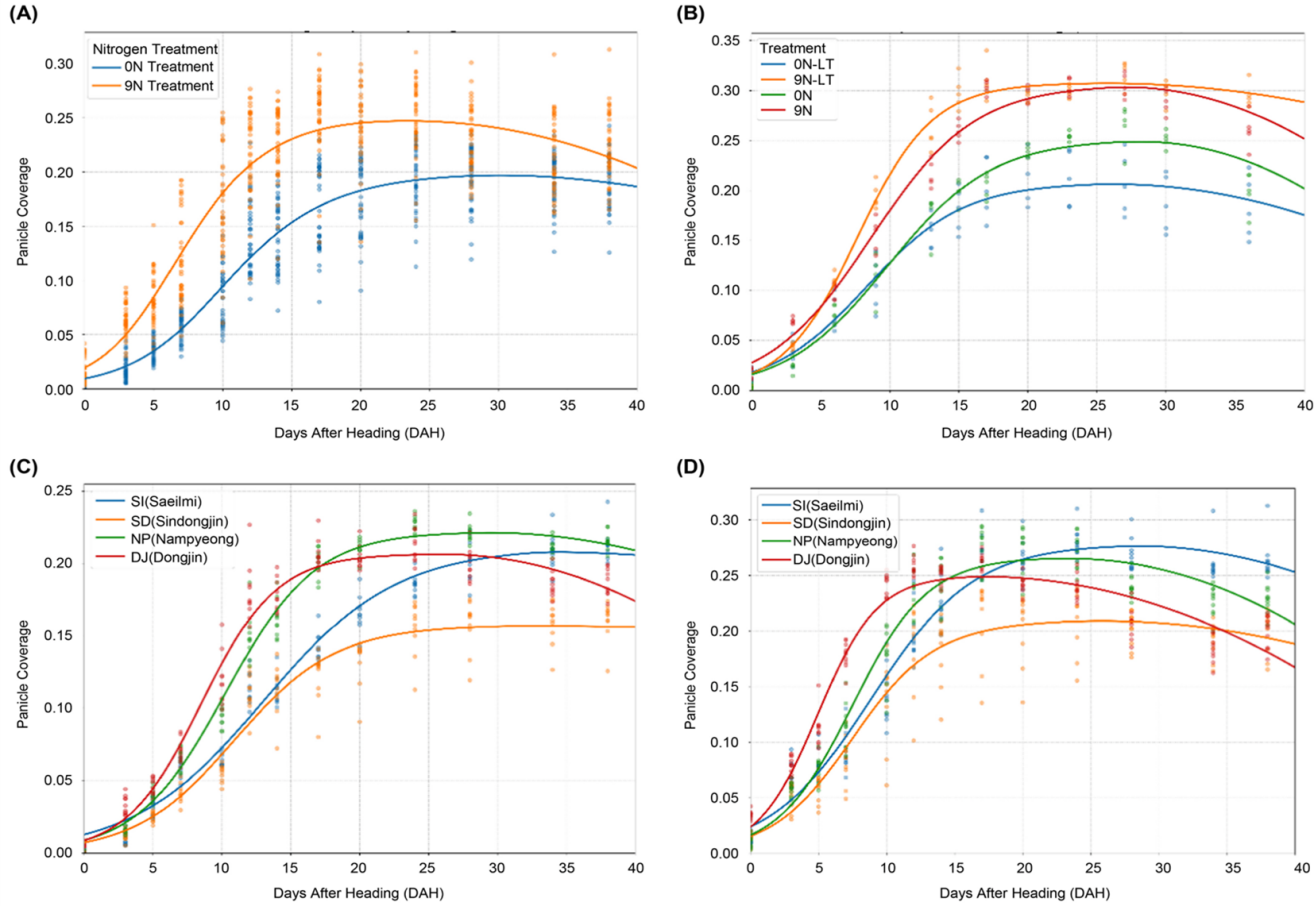
Effects of agricultural treatments on the dynamics of panicle coverage. This figure shows how panicle coverage over time is influenced by different management practices and genetic backgrounds, with each curve representing a fitted piecewise function. **(A)** The effect of nitrogen fertilization on panicle coverage dynamics. **(B)** The effect of late transplantation on panicle coverage dynamics. **(C)** Comparison of panicle coverage dynamics among four different rice varieties under the no-nitrogen (0N) treatment. **(D)** Comparison of panicle coverage dynamics among the same four varieties under a high-nitrogen (9N) fertilization regime.

To investigate the influence of nitrogen, transplantation date, and varietal factors on the panicle coverage dynamics, the time-series changes in the panicle coverage were plotted under different treatment conditions, and the data were fitted to the piecewise function (**Figure 3**).

As confirmed by our ANOVA results, nitrogen treatment had a statistically significant effect on panicle coverage dynamics (**Table 3**), a trend visually represented in **Figure 3A**. Higher nitrogen level (9N) led to a higher maximum panicle coverage (*K*) of 0.2663 compared to 0.2220 under the no-nitrogen control (0N). This suggests that nitrogen promotes panicle growth and development. The quadratic curvature parameter (*a*), however, showed only slight changes with nitrogen treatment, where it remained constant (0.0003) across all treatments.

The effects of the transplantation date and nitrogen availability were also apparent (**Figure 3B**). Late transplantation (LT) under no-nitrogen conditions resulted in a lower maximum panicle coverage (*K*), decreasing from 0.2512 (0N) to 0.2081 (0N-LT). Contrary to the baseline treatment, the transition to the decline phase (*d_1_*) was also earlier in the LT group. With sufficient nitrogen, the adverse effect of late transplantation on *K* was offset, with K values of 0.3060 (9N) and 0.3078 (9N-LT) being nearly identical.

Significant differences in panicle coverage dynamics were observed among cultivars, and these responses were influenced by nitrogen availability, as indicated by the significant main effects of cultivar and nitrogen (**Table 3**) and shown in **Figures 3C, D**. Under the 9N condition, Saeilmi achieved one of the highest *K* values (0.2782) but had a slower growth rate (*g* = 0.2695), whereas Dongjin-1 showed the fastest growth rate (*g* = 0.4606) but a more moderate *K* value (0.2500). These results underscore the intricate interplay of genetics with management practices. These results reveal the complex interplay of nitrogen availability, transplantation timing, and genetic factors in determining the panicle coverage dynamics. The findings emphasize the importance of considering these factors in optimizing nitrogen management and variety selection for rice production.

### 3.5 Correlation between piecewise function parameters and yield components

**Figure 4:**
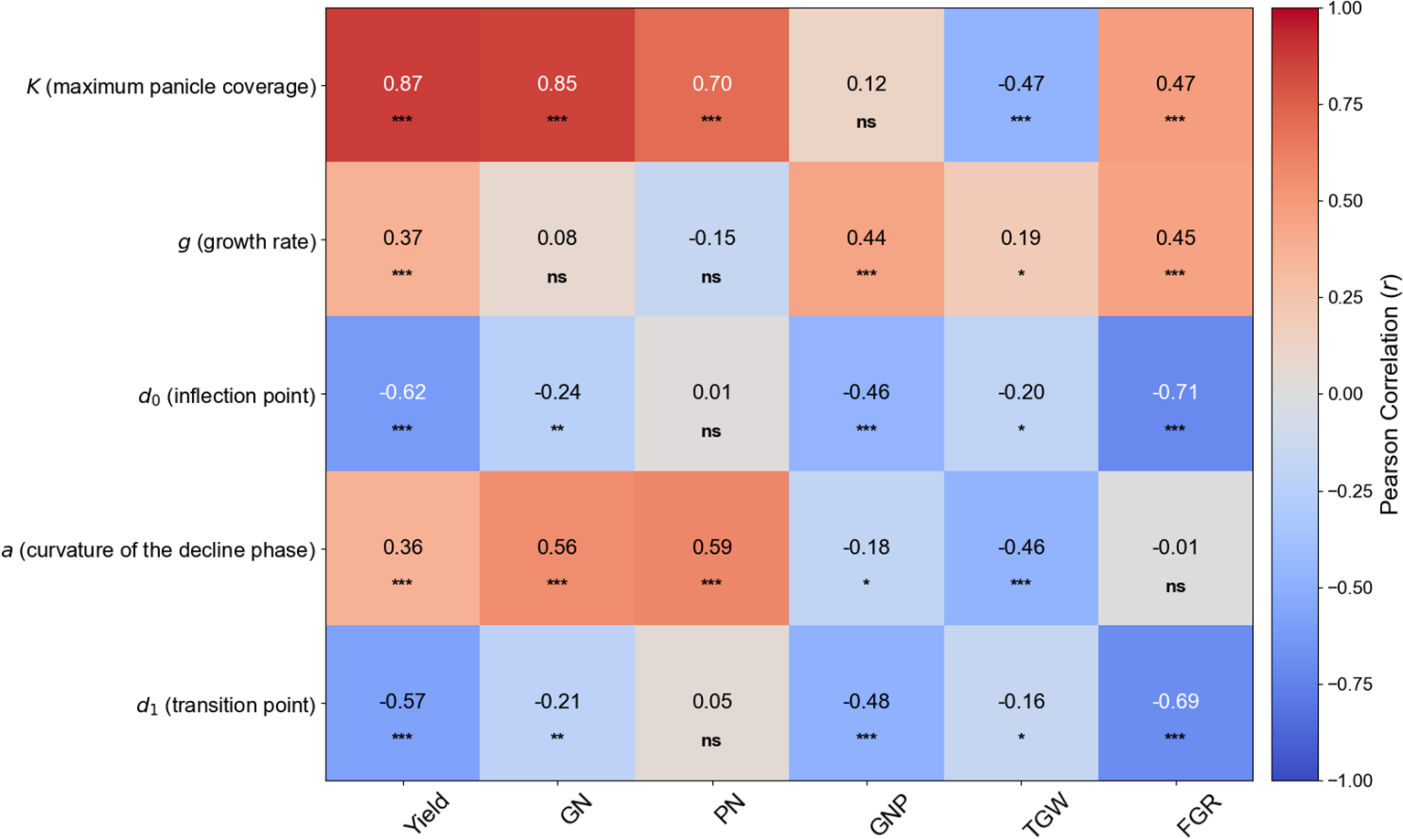
Heatmap of Pearson correlation coefficients between piecewise function parameters and yield components. This heatmap visualizes the linear relationships between the five dynamic parameters (K, g, d_0_, a, and d_1_) and six key yield components (Yield, GN, Grain Number; PN, Panicle Number; GNP, Grains per Panicle; TGW, 1000-Grain Weight; FGR, Filled Grain Ratio). Red cells indicate a positive correlation, while blue cells indicate a negative correlation. The intensity of the color corresponds to the strength of the correlation, with the correlation coefficient (r) value displayed in each cell. Asterisks denote statistical significance levels: *p < 0.05, **p < 0.01, and ***p < 0.001. ‘ns’ indicates a non-significant correlation.

A Pearson correlation analysis was performed to investigate the linear relationship between the five parameters derived from the piecewise function (*K*, *g*, *d_0_*, *a*, and *d_1_*) and the key yield components (**Figure 4**). The analysis identified several significant correlations.

Among the parameters, *K* (maximum panicle coverage) showed the strongest positive correlation with Yield (*r* = 0.87, *p* < 0.001), GN (grain number) (*r* = 0.85, *p* < 0.001), and PN (panicle number) (*r* = 0.70, *p* < 0.001), highlighting its dominant role in determining final crop yield. Interestingly, *K* displayed a significant negative correlation with TGW (1000-grain weight) (*r* = -0.47, *p* < 0.001).

The timing parameters also revealed strong relationships. The time of maximum growth rate (*d_0_*) was strongly negatively correlated with Yield (*r* = -0.62, *p* < 0.001) and especially with FGR (filled grain ratio) (*r* = -0.71, *p* < 0.001). Similarly, the transition point to the decline phase (*d_1_*) showed a strong negative correlation with Yield (r = -0.57, p < 0.001) and FGR (*r* = -0.69, *p* < 0.001). Taken together, these findings underscore the intricate interplay between genetic factors (cultivar) and management practices (nitrogen, transplantation timing) in determining panicle coverage dynamics, emphasizing the importance of an integrated approach for optimizing rice production.

### 3.6 Regression analysis of yield components using models evaluated with LOOCV

Regression plots illustrated the predictive performance of the four models (PLSR, RFR, GBR, and XGBR) in predicting the yield and yield components based on the five parameters derived from the piecewise function (**Supplementary Figure S6**). The models were evaluated using LOOCV; each plot shows the relationship between the actual (x-axis) and predicted values (y-axis). The red diagonal line represents the ideal 1:1 relationship (perfect prediction), and the blue line represents the regression line fitted to the data.

The results demonstrate different accuracies depending on the model and yield component. RFR and XGBR showed high predictive performance for Yield and GN, with points clustered closely around the 1:1 line, indicating good agreement between the predicted and observed values. PLSR and GBR also showed reasonably good predictions for Yield and GN, although with slightly greater scatter. For PN, all models showed moderate prediction accuracy, with RFR and XGBR performing marginally better than PLSR and GBR. In contrast, the predictions for GNP and TGW showed lower accuracy across all models, as evidenced by the increased scatter around the 1:1 line. This reflects a greater inherent variability in these traits, in addition to limitations in the ability of the models to capture them based solely on the piecewise function parameters. The FGR predictions showed moderate accuracy, with RFR and XGBR again demonstrating slightly better performances than those of PLSR and GBR.

### 3.7 Coefficient of determination and RMSE evaluation of yield estimation models

**Table 4** summarizes the predictive performances of the four regression models (PLSR, RFR, GBR, and XGBR) based on the R² and RMSE values for the yield and yield components: GN, PN, GNP, TGW, and FGR.

**Table 4:** Predictive performance of machine learning models for yield and its components.

Yield components	Metric	PLSR	XGBR	RFR	GBR
Yield	R^2^	0.82	0.89	0.89	0.88
GN		0.75	0.82	0.87	0.87
PN		0.61	0.66	0.80	0.80
GNP		0.21	0.27	0.39	0.40
TGW		0.44	0.67	0.70	0.63
FGR		0.57	0.71	0.73	0.73
Yield (kg/ha)	RMSE	75.69	61.16	60.83	61.46
GN (number/m^2^)		3521.43	2943.32	2527.82	2526.56
PN (number/m^2^)		53.86	49.91	38.69	38.05
GNP (GN/PN)		9.93	9.52	8.72	8.65
TGW (g)		1.91	1.46	1.40	1.53
FGR (%)		0.06	0.05	0.05	0.05

The coefficient of determination (R^2^) and root mean squared error (RMSE) are shown for four regression models evaluated using leave-one-out cross-validation (LOOCV).

Regarding the R² values, RFR and XGBR consistently demonstrated superior performance for the key components, such as Yield (R² = 0.890 for both) and GN (R² = 0.870 and 0.820, respectively). GBR also achieved strong results for Yield, GN, and PN, with its performance comparable to that of RFR for these components. PLSR performed relatively well for Yield (R² = 0.820) and GN (R² = 0.750) but underperformed in components such as the GNP (R² = 0.210) and TGW (R² = 0.440), highlighting its limitations in capturing complex, nonlinear relationships.

Considering the RMSE values, RFR and XGBR recorded lower RMSE values for Yield (RMSE = 60.83 and 61.16, respectively) and GN (RMSE = 2527.82 and 2943.32, respectively), indicating their reliability in reducing the prediction errors. Conversely, PLSR displayed higher RMSE values across most components, particularly GN (RMSE = 3521.43) and Yield (RMSE = 75.69), reaffirming its limited predictive capability when compared with that of the nonlinear models. GBR generally performed similarly to XGBR in terms of the RMSE, particularly for Yield, GN, and PN.

These results emphasize the strengths of nonlinear models such as RFR and XGBR in handling complex traits and achieving better prediction accuracy for the yield components. However, traits such as the GNP and TGW consistently recorded lower R² values and higher RMSE across all models, suggesting the inherent difficulty in predicting these components with the available data.

**Figure 5:**
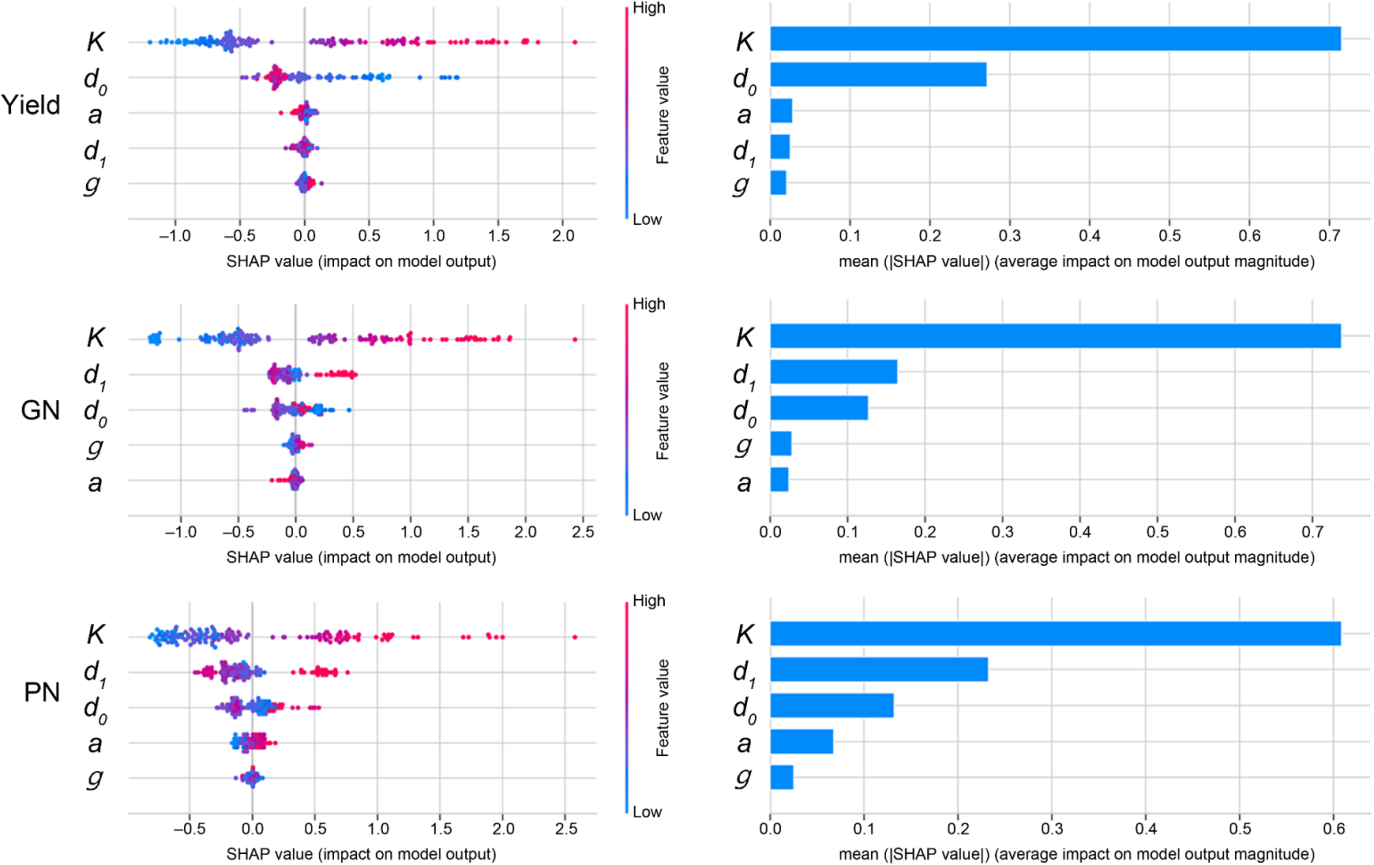
Feature importance analysis for yield prediction models using SHAP (SHapley Additive exPlanations). This figure details the contribution of each piecewise function parameter to the predictions of the Random Forest Regressor (RFR) model for Yield, Grain Number (GN), and Panicle Number (PN). For each yield component, the left plot is a SHAP summary plot, where each dot is a single data point. The color of the dot represents the feature’s value (red for high, blue for low), and its position on the x-axis indicates its impact on the model’s output (positive or negative). The right bar plot ranks the features by their mean absolute SHAP value, indicating their overall importance to the model’s prediction.

**Figure 5**; **Supplementary Figure S7** presents the SHapley Additive exPlanations (SHAP) value analysis for the RFR model, detailing the contribution of each parameter in the piecewise function (*K*, *g*, *d_0_*, *a*, *d_1_*) to the prediction of the yield and yield components. Each point on the plot represents a single data point, with its position on the x-axis indicating the SHAP value (effect on the model output) and its color representing the feature value (red for high, blue for low).

Regarding the yield prediction, *K* (maximum panicle coverage) showed the greatest mean absolute SHAP value and therefore the most significant overall influence, with high values of *K* (red points) consistently associated with positive SHAP values (increased yield prediction); *d_0_* (time of maximum growth rate) was also found to be essential for yield prediction.

Regarding the GN, *K* was again the dominant predictor, followed by *d_0_* and *d_1_*, whereas *K* and *d_1_* were the most important for the PN. The GNP was the most affected by *d_1_* and *K*, whereas the TGW was the most influenced by *K*, *d_1_*, and *d_0_*. The parameters *d_1_* and *d_0_* had the strongest influence on the FGR, with a higher value of *d_1_* (a delayed transition to the decline phase) and a lower value of *d_0_* (delayed onset of the maximum growth rate) generally associated with a higher FGR. The growth rate (*g*) and curvature of the decline (*a*) consistently showed lesser importance across all yield components.

This SHAP analysis confirmed that the parameters derived from the piecewise function, particularly *K*, *d_0_*, and *d_1_*, provide valuable insights into the factors driving the yield and its components. The visual representation of the feature importance facilitates a more nuanced understanding of the predictions made by the model than a simple examination of the overall model performance metrics.

## 4 Discussion

This study demonstrated the effectiveness of a deep learning-based approach integrating high-resolution RGB imagery, semantic segmentation, and time-series analysis for accurately monitoring the rice panicle coverage and predicting the yield components. The framework achieves significant improvements in accuracy and efficiency relative to traditional methods, highlighting its potential for advancing precision agricultural practices in rice production. Specifically, the strong correlation between the predicted and observed values (**Table 4**; **Supplementary Figure S6**) underscores the practical applicability of this technology. Furthermore, the ability to perform these analyses using readily available RGB imagery, rather than specialized equipment, increases the accessibility and potential for widespread adoption of this approach.

Deep learning models, particularly CNNs, are adept at extracting complex features from high-resolution images, effectively addressing challenges such as partial occlusion and variable lighting conditions that often hinder traditional image processing techniques (Lin et al., 2023; Mohammadzadeh Babr et al., 2022; Lu et al., 2024). Our evaluations confirmed this, showing robust performances across diverse environmental conditions (**Table 2**). Notably, the high performance of DeepLabv3+ is consistent with previous studies that have also identified it as a robust model for rice panicle segmentation (Wang et al., 2021). LinkNet also achieved a comparable mIoU to DeepLabv3+ in our experiments, suggesting its suitability for this application.

However, we acknowledge that this level of performance (mIoU < 0.85) indicates room for further optimization. This limitation is likely attributable to the inherent complexity of the target objects and the challenging field conditions. First, severe occlusion is unavoidable as rice panicles grow and overlap with each other and with leaves in a dense canopy, making it fundamentally difficult for any model to delineate precise pixel-level boundaries. Second, uncontrolled lighting conditions, such as shadows and direct sunlight, can alter the appearance of panicles and obscure their features obscure their features (Lin et al., 2023). Lastly, the texture and color of panicles can become similar to those of senescing leaves in later growth stages, potentially confusing the model.

Nevertheless, it is crucial to interpret this segmentation performance within the context of our study’s primary objective: predicting yield components from the temporal dynamics of panicle coverage. The results demonstrate that our framework, even with an mIoU of 0.82, was sufficiently robust to capture the overall trend of panicle development. The high predictive power of the final yield models (R² up to 0.89) strongly supports this, suggesting that capturing the holistic temporal pattern of the canopy is more critical for yield prediction than achieving perfect segmentation of every individual panicle.

While the selected models like DeepLabv3+ and LinkNet provided robust performance for this study’s objectives, we acknowledge that the field of deep learning is rapidly evolving. Future research should therefore focus on improving segmentation accuracy to an even higher standard. A critical next step would be to significantly expand the current dataset to include more diverse genetic backgrounds, environmental conditions, and growth stages. The automated image acquisition platform based on a fixed tower, as implemented in this study, offers an efficient and direct pathway for building the large-scale, longitudinal datasets required to effectively train and validate more advanced architectures. Furthermore, exploring these advanced architectures, such as Vision Transformers (ViT) which have shown promise in handling occlusion (Lu et al., 2024), will be a key priority.

While conventional single-time-point analyses might offer a snapshot correlation, for instance, between panicle number and coverage at the heading stage, they cannot capture the temporal dynamics of grain filling and senescence. The proposed framework, by contrast, not only estimates maximum coverage (*K*) but also quantifies the rates of growth (*g*) and decline (*a*). This allows for a deeper understanding of how the *entire* developmental trajectory, including the speed of maturation and senescence, impacts final yield components like the filled grain ratio (FGR)—an insight largely inaccessible through static measurements.

This study revealed that dynamic parameters derived from time-series panicle coverage are powerful predictors for rice yield. The maximum panicle coverage (*K*) emerged as the strongest predictor for Yield and GN (*r* = 0.87 and 0.85, respectively). This is physiologically sound, as a greater panicle area allows for a higher number of spikelets and increased light interception, ultimately boosting photosynthetic capacity and assimilate production, which aligns with previous findings on the importance of canopy architecture (Gu et al., 2018; Ji et al., 2023). Interestingly, *K* showed a moderate negative correlation with TGW (*r* = -0.47), which can be interpreted as the well-known “yield component compensation effect”. This suggests that when a higher number of grains is secured per unit area, the photosynthates distributed to each grain become relatively limited, leading to a tendency for reduced individual grain weight.

Critically, the introduction of a piecewise function to model the temporal dynamics of panicle coverage provides valuable insights into rice growth and development, allowing us to capture the dynamic processes that influence yield. The strong negative correlations between timing parameters (*d_0_*, *d_1_*) and FGR (*r* = -0.71 and -0.69, respectively) provide critical insights. These results imply that a faster progression to the peak growth and senescence stages is beneficial for grain filling. This could be because rapid panicle development allows the critical grain-filling period to occur under optimal weather conditions, avoiding late-season stresses like high temperatures or insufficient solar radiation that might otherwise hinder full grain development. This highlights the potential of using temporal dynamic parameters to assess the adaptation of the crop to environmental conditions.

Furthermore, the parameters ‘*g*’ (growth rate) and ‘*a*’ (decline rate) provide additional physiological insights. The growth rate ‘g’ likely reflects the initial vigor and uniformity of panicle exsertion, with a higher ‘g’ value indicating efficient nutrient translocation at the beginning of the reproductive stage. More intriguingly, the decline rate ‘*a*’ can be interpreted as a proxy for the grain-filling process. As grains successfully fill and accumulate weight, the panicles begin to droop, which is a key visual indicator of a heavy and well-developing sink. This physical change in canopy architecture, specifically the change in panicle angle, reduces the panicle area visible from the nadir-view camera. Therefore, a higher value for ‘*a*’ may not represent degradation, but rather the positive outcome of effective assimilate partitioning that leads to heavy grains and successful ripening.

The results also demonstrated the significant influence of environmental and genetic factors on panicle coverage dynamics (e.g., Yang et al., 2022), as statistically confirmed by our analysis of variance (**Supplementary Table 3**). As shown in **Figure 3**, nitrogen fertilization levels, transplantation dates, and varietal differences had a measurable statistically significant effect on the parameters of the piecewise function, ultimately affecting the yield components. This underscores the need for tailored management practices that account for these interacting factors.

However, this study has several limitations. First, because the data were collected over a relatively short period and from a single experimental location, it may not adequately reflect the variability introduced by various environmental factors such as weather, pests, and diseases. Future research should consider this variability through multi-environment and long-term studies. Second, because only RGB images were used, additional research is needed to integrate multispectral and thermal imaging to analyze the physiological traits and stress responses (Gitelson et al., 1996; Dorigo et al., 2007; Çolak et al., 2015; Park et al., 2021). Additionally, 3D point cloud data, derived from drone imagery, can provide comprehensive information on the canopy structure and panicle architecture, potentially improving the accuracy of the yield component predictions (Wu et al., 2022; Song et al., 2024). Lastly, the model utilized in this study may be optimized for specific varieties and cultivation conditions, requiring further research to validate its generalization performance across diverse production systems.

Beyond precision agriculture, this panicle coverage-based framework holds promise for plant breeding applications. Drone-based image analysis can facilitate large-scale phenotyping and the identification of superior varieties with enhanced nitrogen use efficiency or stress tolerance (Zhang and Kovacs, 2012; Guan et al., 2019; Bak et al., 2023). Specifically, the ability to rapidly and non-destructively estimate parameters such as *K*, *g*, *d_0_*, and *d_1_* can accelerate the selection of genotypes with desirable growth characteristics.

In conclusion, this study provides a robust and adaptable framework for image-based rice phenotyping, with the potential to significantly improve both agricultural management and crop improvement efforts. By combining deep learning with time-series analysis, the proposed framework serves as a powerful tool for understanding and predicting rice yield, paving the way for more sustainable and efficient rice production.

## 5 Conclusions

This study established and validated a deep learning-based framework for accurate rice panicle segmentation and yield component prediction using time-series RGB imagery. The combination of semantic segmentation (particularly with DeepLabv3+ and LinkNet models) and a piecewise function to characterize the panicle coverage dynamics demonstrated high efficacy. The maximum panicle coverage (*K*) and time of maximum growth rate (*d_0_*) derived from the piecewise function were the key predictors of the yield and yield components. Nonlinear regression models (RFR and XGBR) exhibited superior predictive performance relative to PLSR. The proposed framework offers a practical and easy-to-use approach for high-throughput phenotyping of rice, with significant potential for application to both precision agriculture (optimizing nitrogen management and planting strategies) and plant breeding (by accelerating the evaluation and selection of superior genotypes). Future research will focus on expanding the framework to incorporate additional environmental factors and imaging modalities and validating the approach across multiple locations and growing seasons.

## Data Availability

The raw data supporting the conclusions of this article will be made available by the authors, without undue reservation.

## Supplementary material

Supplementary Figure 1 Image acquisition site and equipment. **(A)** An aerial view of the experimental fields at the National Institute of Crop Science in Wanju-gun, Republic of Korea. **(B)** The fixed tower-mounted camera used for time-series imaging. **(C)** The handheld camera used for acquiring images for model training and validation.

Supplementary Figure 2 Architectures of semantic-segmentation networks evaluated in this study. **(A)** U-Net, **(B)** LinkNet, **(C)** PSPNet, **(D)** FPN, and **(E)** DeepLabv3+; each architecture uses a distinct approach to feature extraction and processing.

Supplementary Figure 3 Representative time-series RGB images of rice canopies under different experimental treatments in 2023 **(A)** and 2024 **(B)**. Images were taken on various dates after transplantation, as indicated on the left. Treatments include different nitrogen-fertilization levels (0N, 9N, 18N), late transplantation (LT), and different cultivars (NP: Nampyeong, DJ: Dongjin-1, SD: Shindongjin, SI: Saeilmi) under both field and soil-bin conditions. The “F” and “S” prefixes denote field and soil-bin experiments, respectively. The number before the hyphen represents the year.

Supplementary Figure 4 Model Training and Validation Curves. The plots show training loss, validation loss, and validation accuracy over 200 epochs for all five evaluated semantic segmentation models, each combined with ResNet-50 and ResNet-101 backbones. The consistent decrease in loss and stabilization of accuracy indicate successful model convergence.

Supplementary Figure 5 Qualitative comparison of segmentation results from different models. The representative input images (“Image”), ground truth segmentations (“GT”), and segmentation outputs of U-Net, LinkNet, PSPNet, FPN, and DeepLabv3+ (all using the ResNet-101 backbone) are shown.

Supplementary Figure 6 Detailed Regression Analysis for Yield Prediction. Regression plots compare the actual and predicted values for all yield and yield components using the four regression models (PLSR, RFR, GBR, and XGBR). Each plot shows the relationship between actual (x-axis) and predicted values (y-axis), with the red line indicating a perfect 1:1 relationship and the blue line representing the fitted regression.

Supplementary Figure 7 SHAP analysis for additional yield components. The figure shows the SHAP (SHapley Additive exPlanations) value analysis for the Random Forest Regressor (RFR) model, detailing the contribution of each piecewise function parameter (*K, g, d_0_, d_1_*, and *a* ​) to the prediction of the number of grains per panicle (GNP), 1000-grain weight (TGW), and filled grain ratio (FGR). For each component, the left plot is a SHAP summary plot where each point represents a single observation, the x-axis indicates the impact on model output, and the color represents the feature value (high=red, low=blue). The right bar plot ranks the features by their mean absolute SHAP value, indicating their overall importance to the model’s prediction.

Supplementary Table 1 Layer architecture of ResNet backbone networks.
