# Supplementary figures and images for: Deep learning-based semantic segmentation for rice yield estimation by analyzing the dynamic change of panicle coverage
Source: Front Plant Sci. 2025 Aug 14;16:1611653. doi: 10.3389/fpls.2025.1611653 (PMC12390994; doi:10.3389/fpls.2025.1611653)

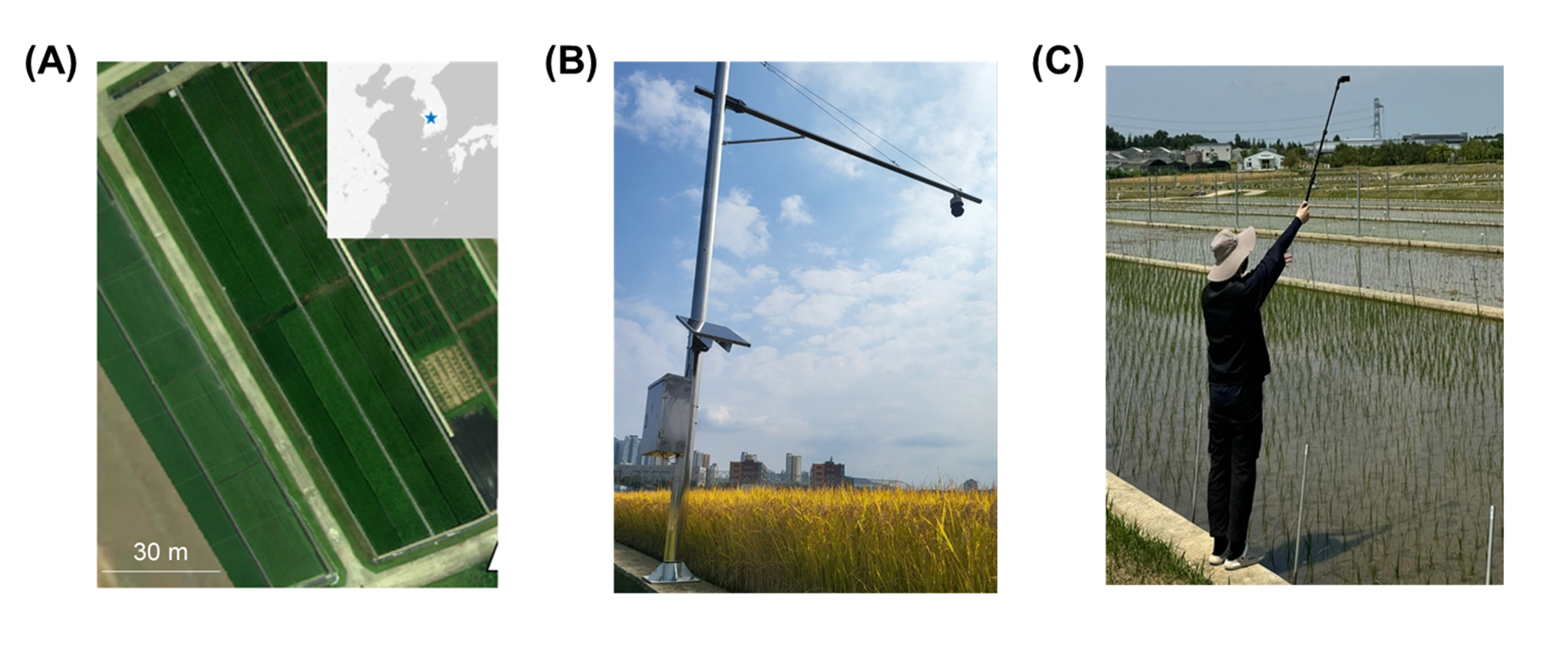

Supplement: Supplementary Figure 1 — Image acquisition site and equipment. (A) An aerial view of the experimental fields at the National Institute of Crop Science in Wanju-gun, Republic of Korea. (B) The fixed tower-mounted camera used for time-series imaging. (C) The handheld camera used for acquiring images for model training and validation. [file Image1.tif]

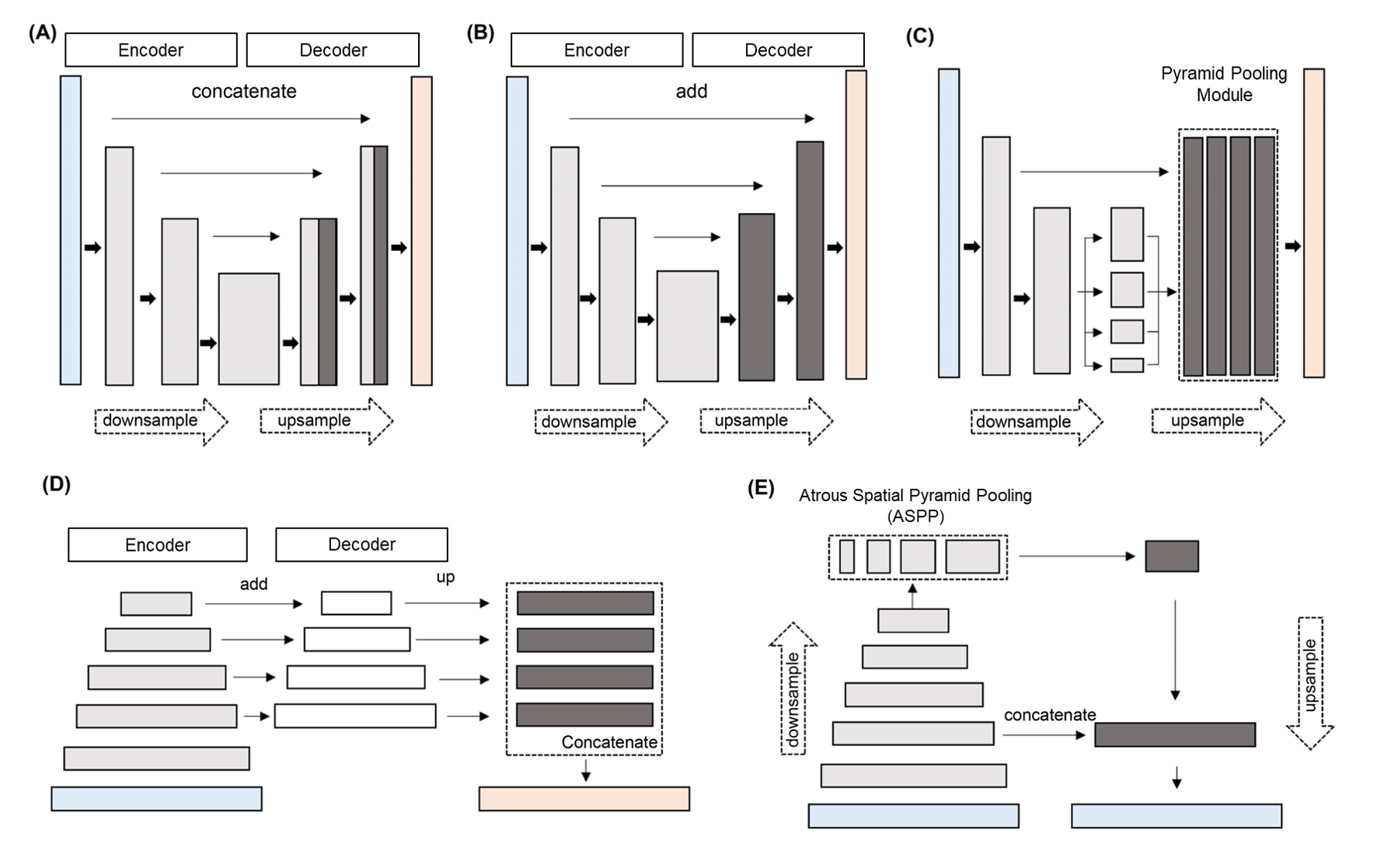

Supplement: Supplementary Figure 2 — Architectures of semantic-segmentation networks evaluated in this study. (A) U-Net, (B) LinkNet, (C) PSPNet, (D) FPN, and (E) DeepLabv3+; each architecture uses a distinct approach to feature extraction and processing. [file Image2.tif]

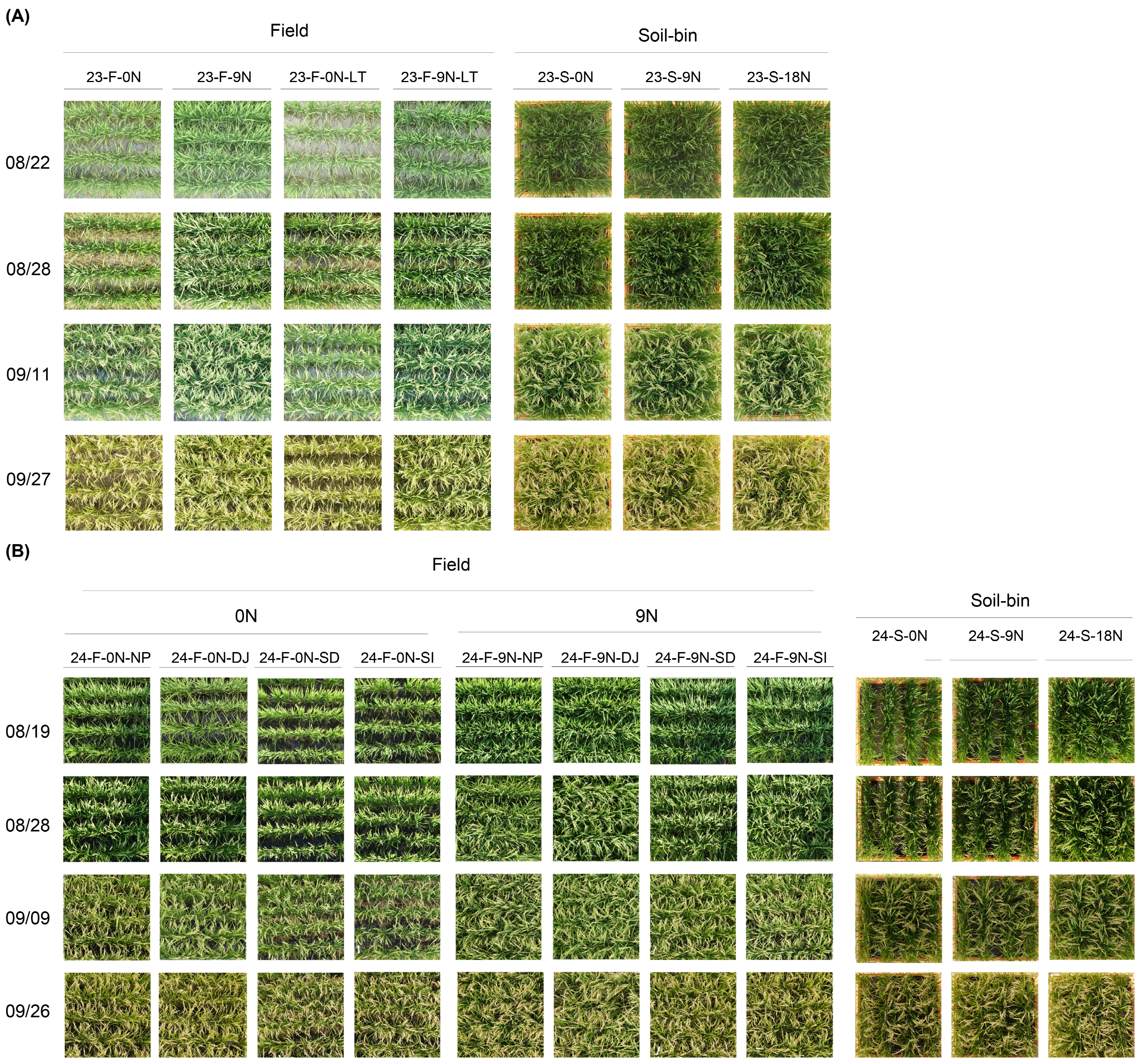

Supplement: Supplementary Figure 3 — Representative time-series RGB images of rice canopies under different experimental treatments in 2023 (A) and 2024 (B). Images were taken on various dates after transplantation, as indicated on the left. Treatments include different nitrogen-fertilization levels (0N, 9N, 18N), late transplantation (LT), and different cultivars (NP: Nampyeong, DJ: Dongjin-1, SD: Shindongjin, SI: Saeilmi) under both field and soil-bin conditions. The “F” and “S” prefixes denote field and soil-bin experiments, respectively. The number before the hyphen represents the year. [file Image3.tif]

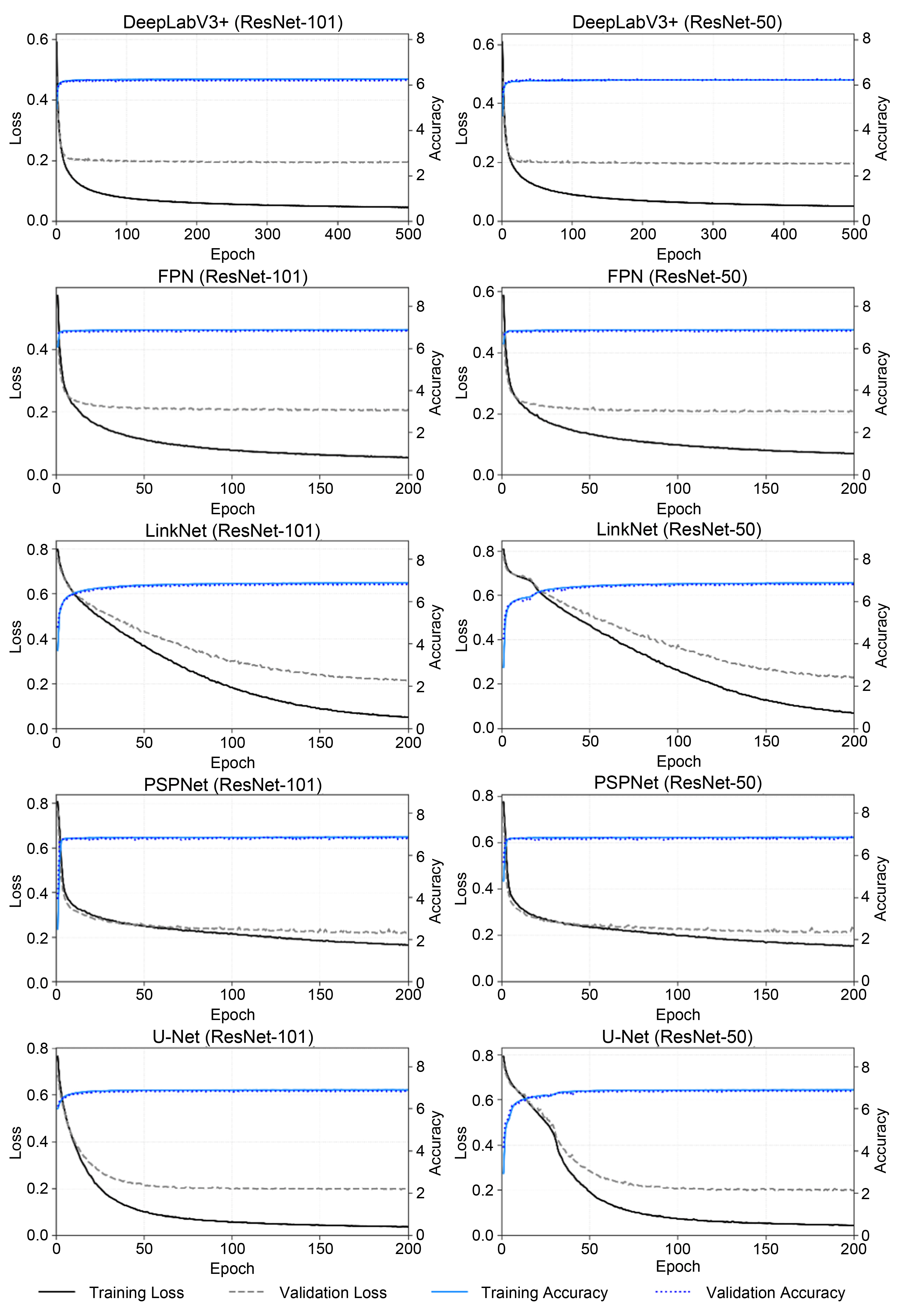

Supplement: Supplementary Figure 4 — Model Training and Validation Curves. The plots show training loss, validation loss, and validation accuracy over 200 epochs for all five evaluated semantic segmentation models, each combined with ResNet-50 and ResNet-101 backbones. The consistent decrease in loss and stabilization of accuracy indicate successful model convergence. [file Image4.tif]

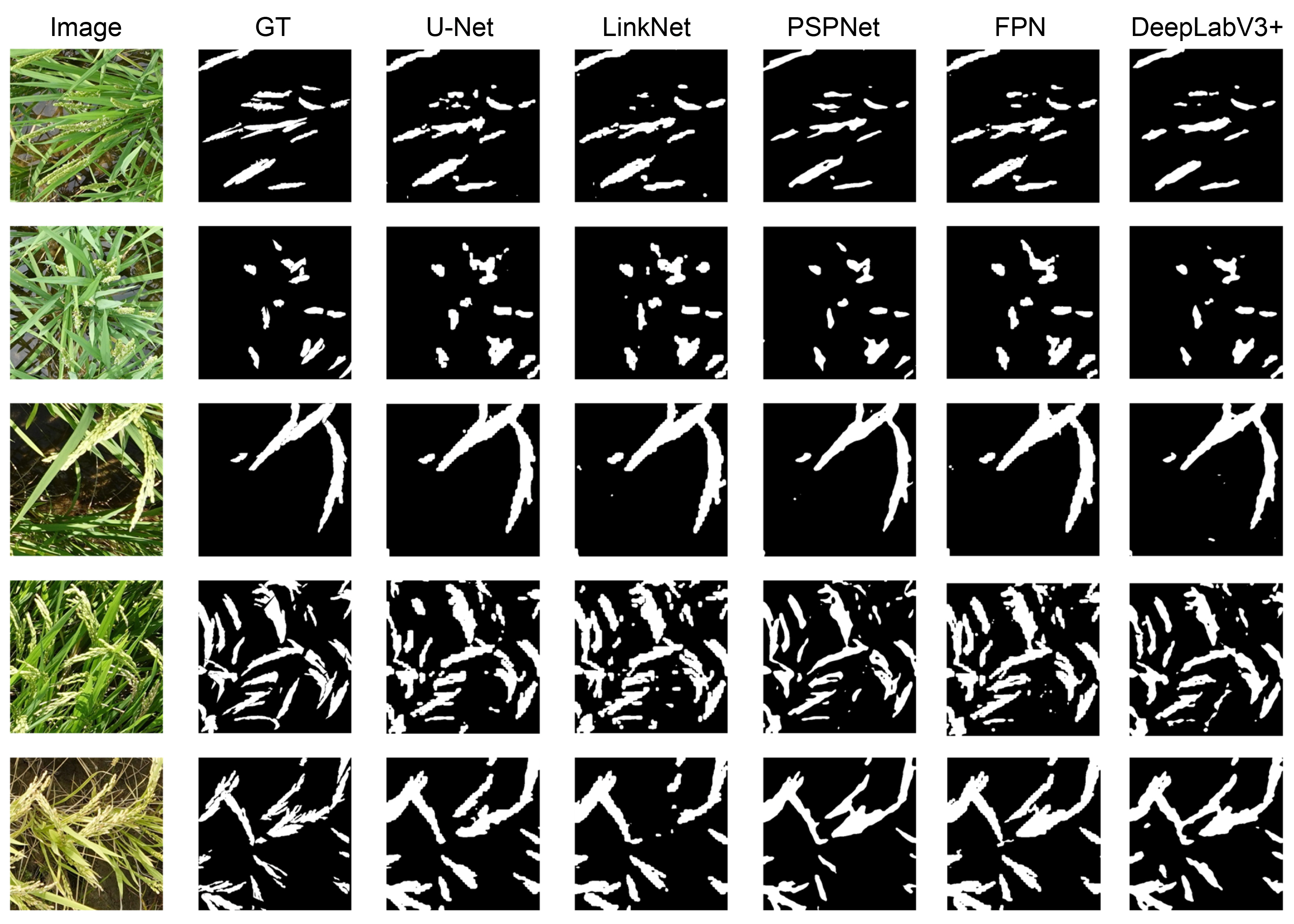

Supplement: Supplementary Figure 5 — Qualitative comparison of segmentation results from different models. The representative input images (“Image”), ground truth segmentations (“GT”), and segmentation outputs of U-Net, LinkNet, PSPNet, FPN, and DeepLabv3+ (all using the ResNet-101 backbone) are shown. [file Image5.tif]

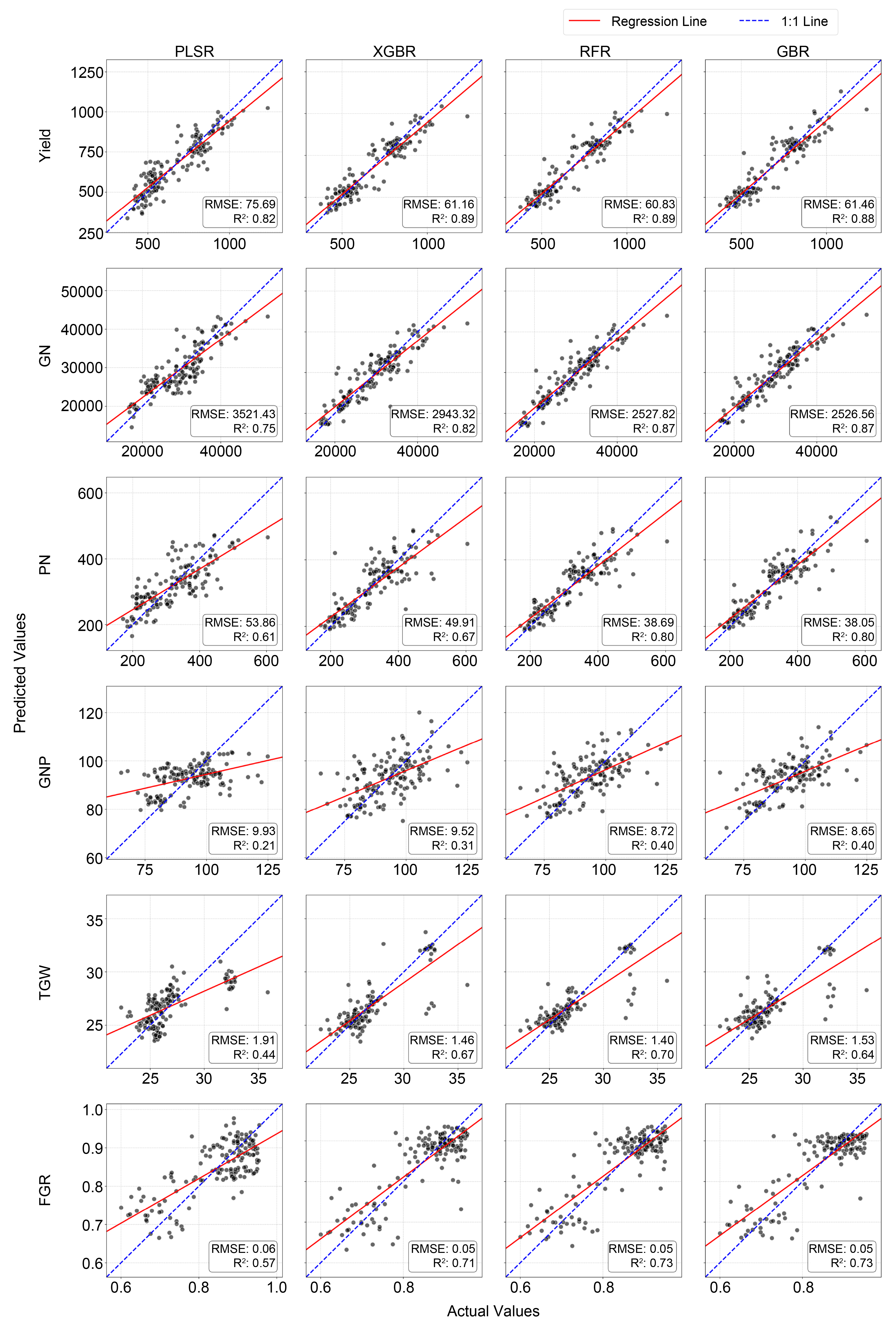

Supplement: Supplementary Figure 6 — Detailed Regression Analysis for Yield Prediction. Regression plots compare the actual and predicted values for all yield and yield components using the four regression models (PLSR, RFR, GBR, and XGBR). Each plot shows the relationship between actual (x-axis) and predicted values (y-axis), with the red line indicating a perfect 1:1 relationship and the blue line representing the fitted regression. [file Image6.tif]

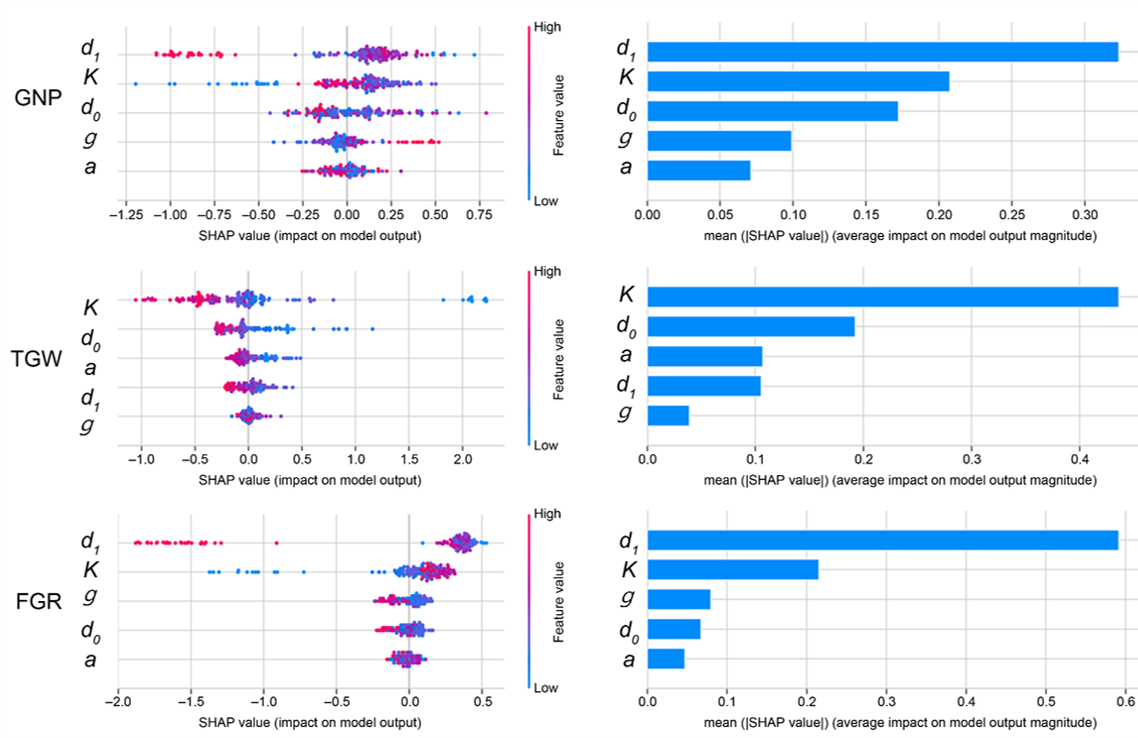

Supplement: Supplementary Figure 7 — SHAP analysis for additional yield components. The figure shows the SHAP (SHapley Additive exPlanations) value analysis for the Random Forest Regressor (RFR) model, detailing the contribution of each piecewise function parameter (K, g, d0, d1 , and a ​) to the prediction of the number of grains per panicle (GNP), 1000-grain weight (TGW), and filled grain ratio (FGR). For each component, the left plot is a SHAP summary plot where each point represents a single observation, the x-axis indicates the impact on model output, and the color represents the feature value (high=red, low=blue). The right bar plot ranks the features by their mean absolute SHAP value, indicating their overall importance to the model’s prediction. [file Image7.tif]
