# Supplementary material for: Deep learning-based semantic segmentation for rice yield estimation by analyzing the dynamic change of panicle coverage
Source: Front Plant Sci. 2025 Aug 14;16:1611653. doi: 10.3389/fpls.2025.1611653 (PMC12390994; doi:10.3389/fpls.2025.1611653)
Supplement: Supplementary Table 1 — Layer architecture of ResNet backbone networks. [file Table1.docx]

**Supplementary Table S1**. Layer architecture of ResNet backbone networks

| **Layer name** | **ResNet-50** | **ResNet-101** |
| --- | --- | --- |
| Conv1 | 7×7, 64, stride 2 | 7×7, 64, stride 2 |
| Conv2 | 3×3 max pool, stride 2 | 3×3 max pool, stride 2 |
|  | [1×1, 64] [3×3, 64]  [1×1, 256] × 3 | [1×1, 64] [3×3, 64]  [1×1, 256] × 3 |
| Conv3 | [1×1, 128] [3×3, 128] [1×1, 512] × 4 | [1×1, 256] [3×3, 256] [1×1, 512] × 4 |
| Conv4 | [1×1, 256] [3×3, 256] [1×1, 1024] × 6 | [1×1, 256] [3×3, 256] [1×1, 1024] × 23 |
| Conv5 | [1×1, 512] [3×3, 512] [1×1, 2048] × 3 | [1×1, 1024] [3×3, 1024] [1×1, 2048] × 3 |
| Global | Global average pool | Global average pool |
|  | 1000-d fc, softmax | 1000-d fc, softmax |
| #params | 25.5×10⁶ | 45.5×10⁶ |
| FLOPs | 3.8×10⁹ | 7.6×10⁹ |
